# Clinical effectiveness and potential predictability of omalizumab in patients with difficult-to-treat chronic rhinosinusitis with nasal polyps and asthma based on the noninvasive markers – A real-life prospective study

**DOI:** 10.1016/j.waojou.2022.100702

**Published:** 2022-09-23

**Authors:** Ming Zheng, Yutong Sima, Chengyao Liu, Jinming Zhao, Shan Shao, Xinmao Wang, Yue Wang, Feifei Cao, Wei Xiong, Xiangdong Wang, Luo Zhang

**Affiliations:** a Department of Otolaryngology Head and Neck Surgery, Beijing TongRen Hospital, Capital Medical University, Beijing, China; b Beijing Laboratory of Allergic Diseases and Beijing Key Laboratory of Nasal Diseases, Beijing Institute of Otorhinolaryngology, Beijing, China; c Department of Otolaryngology Head and Neck Surgery, Beijing Youan Hospital, Capital Medical University, Beijing, China; d Respiratory Department, Beijing TongRen Hospital, Capital Medical University, Beijing, China; e Department of Allergy, Beijing TongRen Hospital, Capital Medical University, Beijing, China; f Research Unit of Diagnosis and Treatment of Chronic Nasal Diseases, Chinese Academy of Medical Sciences, Beijing, China

**Keywords:** Chronic rhinosinusitis with nasal polyps, Asthma, Omalizumab, Difficult-to-treat, Receiver operating characteristic curves

## Abstract

**Background:**

Clinical studies on the effectiveness of omalizumab in patients with difficult-to-treat chronic rhinosinusitis with nasal polyps (CRSwNP) and asthma are scarce in China. Moreover, identifying potential biomarkers predicting its efficacy remains a great challenge.

**Methods:**

In this prospective trial, all enrolled patients underwent endoscopic examination, computed tomography, blood tests, etc, and they completed a 22-item sino-nasal outcome test (SNOT-22), visual analogue scale (VAS), and asthma control test (ACT) evaluation, at baseline and after 24-week omalizumab therapy.

**Results:**

Twenty-two patients were finally recruited. Their VAS scores were significantly better including nasal congestion, anterior rhinorrhea, postnasal drip, and loss of smell (*P* < 0.01). Seventeen patients reported a reduction in SNOT-22 scores of ≥8.9 and 19 patients achieved ACT scores >20. The median change in the Lund-MacKay score (LMS) was 6. Both the Lund-Kennedy score (LKS) and nasal polyp score showed significant improvement (*P* < 0.01). Only 3 parameters in the pulmonary function test showed evident amelioration (*P* < 0.05). The eosinophilic CRSwNP and the male subgroups showed better improvements in subjective and objective evaluation. A receiver operating characteristic curve indicated a cutoff value of 17.5 and 16.5 in LMS had the moderate predictive value (AUC = 0.706) for the decline in the SNOT-22 (more than 8.9 points) and reduction in anterior rhinorrhea VAS (more than 2 cm), respectively. A cutoff value of 18.5 in ACT could provide the moderate predictive value (AUC = 0.771) for the reduction of loss of smell VAS (more than 2 cm).

**Conclusions:**

The beneficial effectiveness of omalizumab in the patients with difficult-to-treat CRSwNP and asthma was confirmed. ECRSwNP and male patients were more likely to have positive responses. The multiple cutoff values for the LMS and ACT may serve as useful predictors for improvement acceptable to difficult-to-treat CRSwNP patients.

## Background

Chronic rhinosinusitis (CRS) is a highly prevalent inflammatory disease of the paranasal sinuses in the respiratory system that affects 10.9%, 12%, and 2.1% of the general population in Europe, the United States, and China, respectively.^1^, ^2^, ^3^ According to nasal endoscopy findings, CRS can be further divided into 2 distinct phenotypic subtypes: chronic rhinosinusitis without nasal polyps (CRSsNP) and chronic rhinosinusitis with nasal polyps (CRSwNP). Furthermore, CRS is also divided into eosinophilic CRS (ECRS) and non-eosinophilic CRS (non-ECRS) according to the degree of eosinophilic infiltration in endotypic classification, in which the former affects more than 80% of CRSwNP patients in Europe and America and poses a higher risk of comorbid severe asthma, leading to more serious clinical exacerbation.^4^^,^^5^ In past decades, despite the popularization and promotion of endoscopic sinus surgery (ESS) and maximal anti-inflammatory treatment strategies during the perioperative period, a considerable number of CRSwNP patients still have persistent and prominent bothersome symptoms after standardized therapy based on clinical guidelines.^6^ Meanwhile, CRSwNP patients often require long-term medical treatment, including repeated courses of systemic corticosteroids and antibiotics, to control frequent relapse; of these patients, 20% experience recurrence after 12 months, with 40% after 18 months, and 80% after 12 years, despite initial successful surgery.^7^, ^8^, ^9^ A recent study reported that almost 100% of patients with CRSwNP and concomitant asthma inevitably had disease recurrence during a 5-year follow-up period, even after ESS combined with Draf III and ongoing medical therapy.^10^ Moreover, approximately 10% of CRS patients did not respond well to adequate medical treatment and revision ESS and developed the difficult-to-treat form;^11^ 29% of Chinese patients were diagnosed as difficult-to-treat, and 41.8% of Belgium CRSwNP patients exhibited a recalcitrant status.^12^^,^^13^

Most cases of CRSwNP share the same pathogenesis with severe asthma, which is characterized by T helper 2 (Th2) cell-biased inflammatory reactions, eosinophilia, and immunoglobulin E (IgE) hyperproduction, in which IgE plays a crucial role in stimulating related type 2 inflammatory cells, such as mast cells, basophils and eosinophils.^14^ An increasing number of novel biologics targeting key immunological markers in the inflammatory cascade reaction, such as IgE, IL-4 and IL-5, have been gradually developed. As a humanized anti-IgE monoclonal antibody, omalizumab was approved for moderate-to-severe asthma first in 2003 by the U.S. Food and Drug Administration (FDA), then in 2005 by the European Medicines Agency (EMA), subsequently in 2018 by National Medical Products Administration (NMPA) in China, and then further approved for severe CRSwNP in 2020 by the FDA.^15^ Although mounting evidence has demonstrated that omalizumab seems to be an attractive and beneficial option for treating recalcitrant CRSwNP,^16^^,^^17^ it remains uncertain whether any markers could predict the successful outcome of omalizumab due to a lack of convincing criteria for determining therapeutic effectiveness. Recently, the European Forum for Research and Education in Allergy and Airway Diseases (EUFOREA) steering group recommended multiple standards based on the application of the minimal clinically important difference (MCID), such as the 22-item sino-nasal outcome test (SNOT-22, reduction of ≥8.9), visual analogue scale (VAS, reduction of ≥2), nasal polyp score (NPS, decrease by ≥ 1) and so on, as the indicator for improvement acceptable to refractory CRSwNP patients.^18^

Until now, clinical research on the effectiveness of omalizumab in CRSwNP patients has been scarce in China. The primary objective of this prospective study was to evaluate the real-life effectiveness of omalizumab in Chinese patients with difficult-to-treat CRSwNP. The secondary objective was to identify potential noninvasive indicators for predicting the optimal response to anti-IgE treatment.

## Methods

### Subjects

Consecutive patients with difficult-to-treat CRSwNP and concomitant asthma from the rhinology department of the tertiary university hospital, who were diagnosed on the basis of European Position Paper on Rhinosinusitis and Nasal Polyps (EPOS) guidelines (2012)^11^ and by the respiratory physician based on Global Initiative for Asthma (GINA) guidelines^19^ were recruited in this study. Although all patients had received adequate ESS and maximal medical treatment in the past year, including intranasal corticosteroid therapy combined with up to 2 short courses of antibiotic or systemic corticosteroids, they still had persistent symptoms (nasal congestion, anterior rhinorrhea, postnasal drip, facial pain, or loss of smell) that seriously affected daily life. Considering the high recurrence rate and serious adverse impact on quality of life (QOL), rhinologists suggested omalizumab treatment for these patients. All patients received serum total IgE test, which were between 60 and 635 kU/L. The dose of omalizumab (Novartis Pharma Stein AG, Switzerland) was prescribed based on the total serum IgE levels and body weight (in kilograms) (Table 1). Patients who were pregnant and lactating, younger than 18 years old, had psychological disorders, autoimmune diseases (IgG4-related diseases, granulomatosis with polyangiitis, etc), had received oral corticosteroids within the past 4 weeks, and had ever received monoclonal antibody treatment or immunosuppressive treatment were excluded from this study.

**Table 1:** Demographic characteristics of the subjects.

	Omalizumab	*P* value
Age (y), median (IQR)	44.0 (34.0–51.0)	–
Male/female (n)	14/8	0.201
Weight (kg), median (IQR)	76.0 (62.0–83.5)	–
BMI (kg/m2), median (IQR)	25.6 (21.5–27.6)	–
Smoke, n (%)	1 (4.6)	–
Total IgE (IU/mL), median (IQR)	143.0 (77.8–223.5)	–
sIgE+/sIgE- (n)	12/10	0.670
ECRSwNP/non-ECRSwNP (n)	16/6	0.033∗
NSE+/NSE- (n)	15/7	0.088

IQR, interquartile range; BMI, body mass index; sIgE +, specific IgE positive*;* sIgE -, specific IgE negative; ECRSwNP, eosinophilic chronic rhinosinusitis with nasal polyp; non-ECRSwNP, non-eosinophilic chronic rhinosinusitis with nasal polyp; NSE +, nasal smear examination positive; NSE -, nasal smear examination negative. ∗*P* < 0.05

This study was approved by the ethics committee of the tertiary university hospital and Chinese Clinical Trial Registry, and all patients provided written informed consent before participation.

### Study design

This study was a self-controlled prospective trial conducted from January 2019 to December 2021. All enrolled patients received omalizumab hypodermic injections every 4 weeks/24 weeks in total. Meanwhile, patients were routinely prescribed inhaled corticosteroids for the control of asthma by a respiratory physician. During the whole treatment period, patients were not allowed to use oral corticosteroids, oral or nasal antihistamines, antibiotics, leukotriene receptor antagonists or nasal decongestants.

At baseline and after 24 weeks of treatment, patients were asked to complete the asthma control test (ACT), and underwent computed tomography (CT) test, pulmonary function test, nasal smear eosinophilic test and routine blood test, including peripheral blood eosinophil counts and blood eosinophil percentages. Patients were asked to complete the VAS, SNOT-22 questionnaire, and received nasal endoscopic examinations at baseline, week 8, week 16, and week 24.

### Sinus CT scan, nasal endoscopic examination, and nasal polyp score

Sinus opacification from CT scans was quantified using the Lund-MacKay score (LMS).^20^ The endoscopic examination was assessed by the Lund-Kennedy score (LKS).^21^ Nasal polyp score (NPS) was evaluated according to the 5-point scale (0 = no polyps; 1 = small polyps in the middle meatus not reaching below the inferior border of the middle concha; 2 = polyps reaching below the lower border of the middle turbinate; 3 = large polyps reaching the lower border of the inferior turbinate or polyps medial to the middle concha; 4 = large polyps causing almost complete congestion of the inferior meatus).^22^

### Total immunology E, nasal smear eosinophil count, and routine blood test

The concentrations of serum total IgE and specific IgE (sIgE) were measured using the UniCAP system (Phadia, Uppsala, Sweden) and the EUROBlotMaster system (EUROIMMUN Medizinische Labordiagnostika AG, Lübeck, SH, Germany), respectively. The sIgE included the mixture of willows, poplars, and elms, ragweed, mugwort, *Dermatophagoides pteronyssinus*, *Dermatophagoides farinae*, cat, dog, and *Humulus*). According to the concentration of sIgE higher than 0.70 kU/L (based on the RAST classification), the patients were divided into the sIgE+ (positive) group and the sIgE– (negative) group.

A sample for nasal smear examination (NSE) was collected by scraping the mucous membrane of the surface of the inferior turbinate with a sterile cotton swab. The secretions were then spread onto a glass slide and air-dried. The smears were stained with May-Grünwald-Giemsa stain, and the number of eosinophils per high-power field (HPF) was counted by a trained technician. Eosinophilia was evaluated semiquantitative by the following: level 0 = 0/HPF, level 1 = 1–5/HPF, level 2 = 6–10/HPF, level 3 = 10–20/HPF and level 4 = more than 20/HPF.^23^ NSE result lower than level 1 was considered the NSE– (negative) group, while the others belonged to the NSE+ (positive) group. Peripheral blood eosinophilic data were obtained from routine blood tests. According to the peripheral blood eosinophilic percentage (Eos%), whether higher or lower than 4.27% cited by EPOS,^6^^,^^24^ enrolled CRSwNP patients were divided into ECRSwNP and non-ECRSwNP groups.

### Pulmonary function test

The pulmonary function test (PFT) included forced expiratory volume in 1 s (FEV_1_), forced vital capacity (FVC), FEV_1_/FVC, vital capacity (VC), peak expiratory flow (PEF), maximum expiratory flow at 75% of vital capacity (MEF_75)_, maximum expiratory flow at 50% of vital capacity (MEF_50_), maximum expiratory flow at 25% of vital capacity (MEF_25_) and MMEF_75/25_.

### VAS scores, SNOT-22 scores, and ACT scores

VAS scores were assessed subjectively by patients to evaluate the severity of their diseases on a scale from 0 cm to 10 cm (from not troublesome to extremely troublesome), with symptoms including nasal congestion, anterior rhinorrhea, postnasal drip, and loss of smell. Patients evaluated their symptoms, sleep, and functional and emotional consequences of CRS by the SNOT-22 questionnaire on a 6-category scale ranging from 0 to 5 (from not problematic to extremely problematic).^25^ The ACT questionnaire comprised 5 items that were used to assess daytime and nocturnal asthma symptoms, use of rescue medications and the influence of asthma on daily functioning. Each item includes 5 response options corresponding to a 5-point scale.^26^ Higher total scores indicate better asthma control, and a score less than 20 reflects uncontrolled asthma.^27^

### Statistical analysis

IBM SPSS 23.0 software (IBM Corp., Armonk, NY, USA) and GraphPad Prism 9 (GraphPad Software Inc., LA Jolla, CA, USA) software were used to analyze the data. A 2-sample *t*-test was used to compare age, sex, the sIgE positive ratio, the NSE positive ratio and the ECRSwNP ratio. The Kolmogorov–Smirnov test was used to evaluate the normal distribution of data for continuous variables, while Fisher's exact test was used for categorical data. Results were analyzed by nonparametric statistical tests, and data were expressed as the median and interquartile range (IQR). The CRS symptoms, and nasal endoscopic scores at weeks 8, 16, and 24 compared with self-baseline were evaluated based on the *Friedman* test. The ACT*,* CT scores, and PFT results after week 24 were compared with baseline by the Wilcoxon signed-rank test. The changes in the CRS symptom scores, CT scores, and nasal endoscopic scores in the different groups were analyzed according to the 2-tailed Mann–Whitney *U* test. Bonferroni corrections were applied for the analysis of comparators, and *P* values less than 0.05 were considered statistically significant.

Receiver operating characteristic (ROC) curves were used to predict the patients’ symptom changes after treatment. A total SNOT-22 change of more than 8.9 points, or a VAS reduction of more than 2 cm was considered an improvement acceptable to CRSwNP patients.^18^ The predictive ability (Eos%, Eos count, total IgE, ACT, age, body mass index [BMI], LMS, LKS and NPS) was calculated based on the area under the ROC curve (AUC). AUC values higher than 0.9, 0.7 to 0.9 and 0.5 to 0.7 represent high, moderate, and low accuracy, respectively. The optimal cutoff was determined by the Youden index, which was used to calculate the sensitivity and specificity of predictors.

## Results

### Patient enrollment

A total of 27 patients with difficult-to-treat CRSwNP and concomitant asthma were screened. Of those, 5 patients were excluded from analysis because of incomplete examination results or poor compliance. 22 consecutive patients were finally enrolled in this prospective cohort study, which consisted of 14 male patients and 8 female patients. All patients completed the VAS, ACT, and SNOT-22 questionnaires, nasal endoscopic examination, and NSE test punctually. One patient did not get the CT scan, 1 patient did not complete the routine blood test and 2 patients did not perform the PFT. Based on the results of the specific IgE, NSE and peripheral blood Eos%, there were 12 patients in the sIgE + subgroup vs. 10 patients in the sIgE– subgroup, 15 in the NSE + subgroup vs. 7 in the NSE– subgroup, and 16 ECRSwNP patients vs. 6 non-ECRSwNP patients among the total enrolled patients.

During the whole treatment period, rhinologists recommended that the patients use nasal glucocorticoids regularly, but 17 of 22 patients used nasal glucocorticoids only as needed. After 24 weeks of omalizumab treatment, 16 patients continued subcutaneous omalizumab every 4 weeks, 3 patients underwent revision ESS again, and 3 patients chose conventional medical treatment. We also monitored vital signs, blood analyses and adverse event reports. No patients reported any treatment-related general or local discomfort or adverse events.

### Subjective clinical symptoms outcomes

**Fig. 1:**
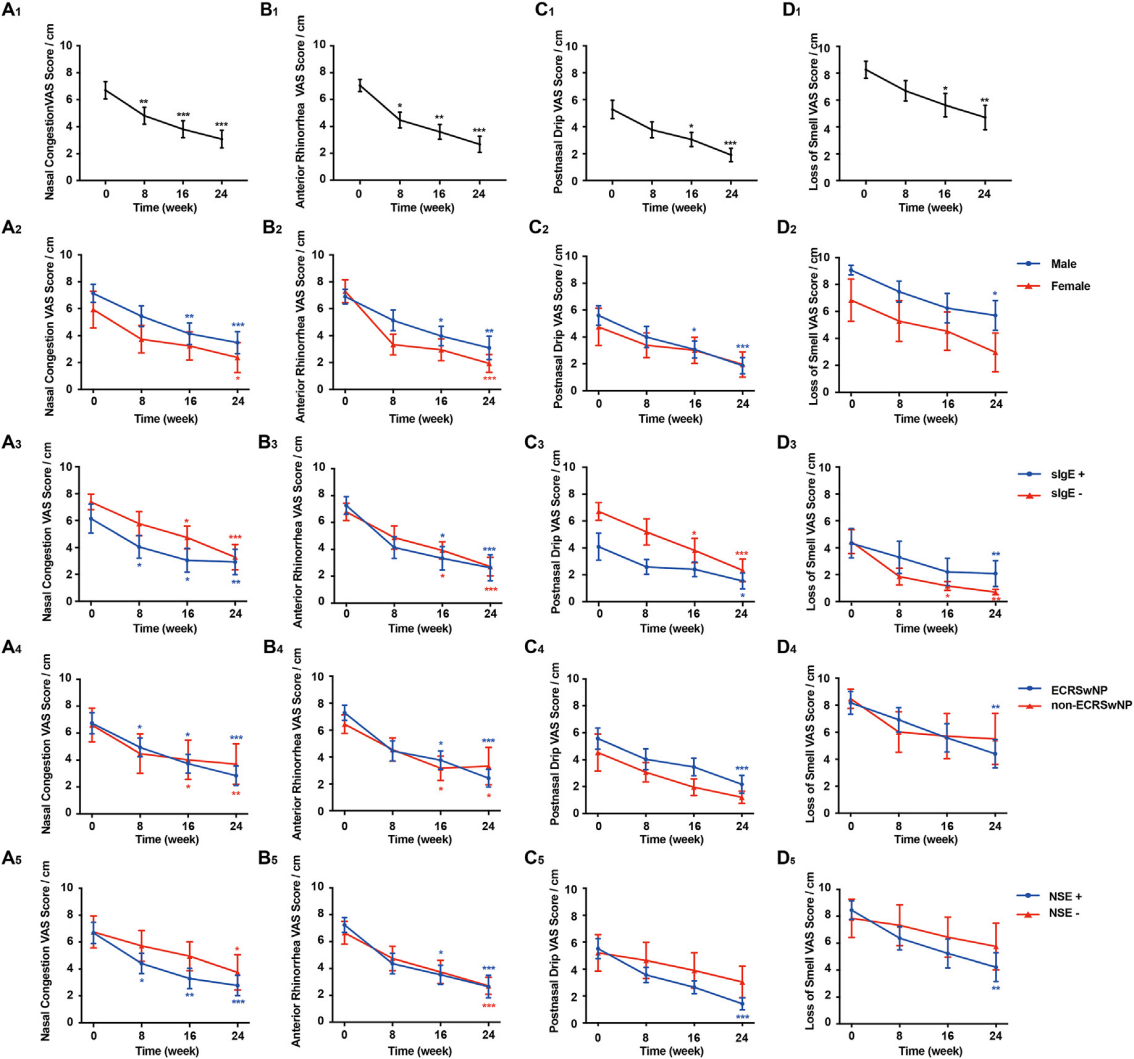
**Mean change in VAS from baseline and after 24-week of omalizumab treatment.** All subjects' mean changes from baseline and after 24 weeks with respect to nasal congestion (A), anterior rhinorrhea (B), postnasal drip (C), and loss of smell (D) during the treatment of omalizumab were shown in A_1_, B_1_, C_1_, and D_1_. The mean changes in the male subjects and female subjects from baseline over the span of weeks are shown in A_2_, B_2_, C_2_, and D_2_. The mean changes in the specific IgE-positive groups and specific IgE-negative groups from baseline over the span of weeks are shown in A_3_, B_3_, C_3_, and D_3_. The mean changes in the ECRSwNP subjects and non-ECRSwNP groups from baseline over the span of weeks are shown in A_4_, B_4_, C_4_, and D_4_. The mean changes in nasal smear eosinophilia-positive groups and nasal smear eosinophilia-negative groups from baseline over the span of weeks are shown in A_5_, B_5_, C_5_, and D_5_. ∗*P* < 0.05. ∗∗*P* < 0.01. ∗∗∗*P* < 0.001. Error bars indicate mean ± SEM.

There was a significant improvement in the VAS score between baseline and week 24, including nasal congestion (*P* < 0.001), anterior rhinorrhea (*P* < 0.001), postnasal drip (*P* < 0.001), and loss of smell (*P* = 0.001) (Fig. 1A_1_–D_1_). Considering the patients’ sex, the nasal congestion, and anterior rhinorrhea showed significant reduction in both subgroups, while the male subgroup showed more observable reduction than that in the female subgroup; the male subgroup showed earlier improvement in nasal congestion and anterior rhinorrhea than that in female subgroup (Fig. 1 A2 and B2); and the postnasal drip and loss of smell at week 24 in the male subgroup showed significant differences compared with the baseline, while the difference was not demonstrated in the female subgroup (Fig. 1C2 and D2). All clinical symptoms were significantly improved in both sIgE+ and sIgE-subgroups (Fig. 1 A3–D3). All symptoms were showed significantly improved in the ECRSwNP subgroup between the baseline and 24-week treatment of omalizumab, but only nasal congestion, and anterior rhinorrhea were significantly improved in the non-ECRSwNP subgroup (Fig. 1 A4–D4). Similarly, the NSE + subgroup in all symptoms showed significant reduction between baseline and week 24; while only *2* symptoms, nasal congestion and anterior rhinorrhea, were significantly improved in the NSE-subgroup (Fig. 1 A5–D5). The changes in clinical symptoms in different subgroups, which were divided based on clinical characteristics, were not statistically significant (see Fig. 1).

**Fig. 2:**
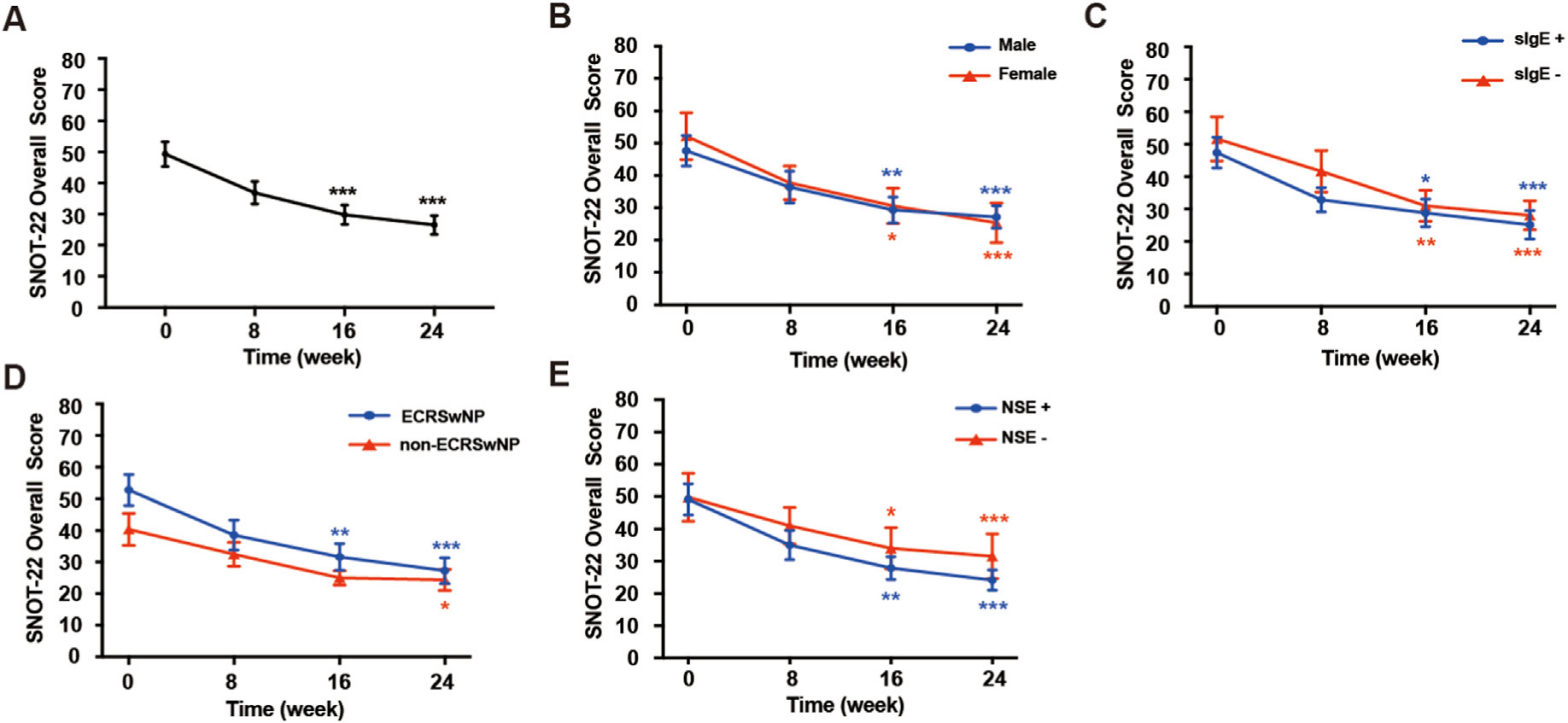
**Mean change in the SNOT-22 score from baseline and after 24-week of omalizumab treatment.** All subjects' mean change in SNOT-22 overall scores (A) from baseline and after 24 weeks. The mean change in SNOT-22 overall scores in the male groups and female groups (B), the specific IgE-positive groups and specific IgE-negative groups (C), the ECRSwNP groups and non-ECRSwNP groups (D) and the nasal smear eosinophilia-positive groups, nasal smear eosinophilia-negative groups (E) from baseline over 24 weeks of treatment with omalizumab. ∗*P* < 0.05. ∗∗*P* < 0.01. Error bars indicate mean ± SEM.

Seventeen of 22 patients (77.27%) reported a reduction in total SNOT-22 scores of more than 8.9 after 24-week treatment of omalizumab. The total SNOT-22 scores showed significant improvement in all patients between baseline and week 24 (*P* < 0.001) (Fig. 2A). According to the evaluation of SNOT-22, all the subgroups showed significant improvement [the male subgroup (*P <* 0.001), the female subgroup (*P <* 0.001), the sIgE + subgroup (*P* < 0.001), the sIgE-subgroup (*P* < 0.001), the ECRSwNP subgroup (*P* < 0.001), non-ECRSwNP subgroup (*P* = 0.031)*,* the NSE + subgroup (*P* < 0.001), and the NSE-subgroup (*P* < 0.001)] (Fig. 2B–E). The ECRSwNP subgroup showed more observable improvement in SNOT-22 than that in the non-ECRSwNP subgroup. The changes in SNOT-22 scores in various subgroups were no significant difference between subgroups, respectively.

The ACT scores of patients showed significant improvement compared with baseline (*P* = 0.012) (Table 2). Notably, in the male/female subgroup, the sIgE + subgroup, the ECRSwNP subgroup, and the NSE ±subgroup, patients’ asthma control scores showed a significant improvement compared with baseline; nineteen of 22 patients (86.36%) reached the asthma control level (ACT scores >20) after 24-week treatment of omalizumab.

**Table 2:** Clinical characteristics of subjects at week 0 and week 24 of treatment.

	Week 0	Week 24	Change	*P*_week 0–24_	*P*_between groups baseline_	*P*_changes between 0–24_
**ACT scores**						
Overall, median (IQR) n = 22	20.00 (15.75–23.00)	22.00 (20.00–25.00)	2.00 (0.00 to 6.00)	0.012∗∗	–	–
Male, median (IQR) n = 14	20.00 (14.50–22.25)	21.00 19.75–25.00)	2.00 (−0.25 to 5.50)	0.020∗	0.815	0.664
Female, median (IQR) n = 8	20.00 (16.00–23.75)	22.00 (21.25–25.00)	2.00 (0.25 to 6.00)	0.034∗		
sIgE +, median (IQR) n = 12	19.50 (13.00–23.75)	22.00 (20.25–25.00)	2.00 (0.25 to 7.50)	0.008∗∗	0.872	0.254
sIgE -, median (IQR) n = 10	21.00 (15.75–22.25)	21.50 (19.00–25.00)	1.50 (−1.0 to 3.75)	0.107		
ECRSwNP, median (IQR) n = 16	18.50 (13.75–22.0)	21.50 (20.00–24.25)	2.00 (0.50 to 6.75)	0.003∗∗	0.083	0.134
non-ECRSwNP, median (IQR) n = 6	23.00 (18.75–24.25)	25.00 (19.50–25.00)	0.50 (−0.25 to 2.00)	0.257		
NSE +, median (IQR) n = 15	20.00 (16.00–22.00)	22.00 (21.00–25.00)	2.00 (−1.00 to 6.00)	0.015∗	0.891	NS
NSE -, median (IQR) n = 7	18.00 (15.00–24.00)	22.00 (20.00–25.00)	2.00 (0.00 to 6.00)	0.042∗		
**Total NPS**						
Overall, median (IQR) n = 22	3.00 (2.00–5.00)	1.50 (0.00–2.25)	1.00 (−3.00 to 0.00)	0.001∗∗	–	–
Male, median (IQR) n = 14	3.50 (2.00–5.25)	1.00 (0.75–2.25)	1.50 (−3.00 to 0.00)	0.005∗∗	0.664	0.525
Female, median (IQR) n = 8	3.00 (1.00–5.00)	2.00 (0.00–3.50)	0.00 (−2.75 to −1.00)	0.041∗		
sIgE +, median (IQR) n = 12	4.00 (1.25–5.75)	1.50 (1.00–4.50)	1.00 (−2.75 to 0.00)	0.017∗	0.628	0.497
sIgE -, median (IQR) n = 10	3.00 (2.00–5.00)	1.50 (0.00–2.00)	1.50 (−3.00 to −0.75)	0.011∗		
ECRSwNP, median (IQR) n = 16	4.50 (1.25–5.75)	1.50 (0.25–2.75)	1.50 (−3.00 to 0.00)	0.005∗∗	0.541	0.858
non-ECRSwNP, median (IQR) n = 6	3.00 (2.00–3.50)	1.50 (0.00–2.75)	1.00 (−2.25 to −0.75)	0.039∗		
NSE +, median (IQR) n = 15	3.00 (2.00–6.00)	1.00 (0.00–3.00)	1.00 (−5.00 to −2.00)	0.005∗∗	0.680	0.945
NSE -, median (IQR) n = 7	3.00 (2.00–5.00)	2.00 (0.00–2.00)	1.00 (−3.00 to 0.00)	0.038∗		
**LMS**						
Overall, median (IQR) n = 21	19.00 (15.50–23.00)	14.00 (7.50–18.00)	6.00 (−11.00 to 0.50)	0.002∗∗	–	–
Male, median (IQR) n = 14	19.0 (16.75–24.00)	14.5 (8.75–18.00)	5.5 (−11.25 to 0.25)	0.016∗	0.224	0.913
Female, median (IQR) n = 7	14.00 (11.00–23.00)	12.00 (5.00–16.00)	6.00 (−8.00 to 1.00)	0.063		
sIgE +, median (IQR) n = 11	19.00 (15.00–23.00)	14.00 (7.00–18.00)	5.00 (−10.00 to 0.00)	0.022∗	0.552	0.552
sIgE -, median (IQR) n = 10	19.00 (16.25–23.25)	12.50 (7.75–18.75)	6.50 (−11.75 to 1.50)	0.041∗		
ECRSwNP, median (IQR) n = 15	19.00 (15.00–23.00)	12.00 (8.00–17.00)	6.00 (−11.00 to 0.00)	0.006∗∗	0.970	0.733
non-ECRSwNP, median (IQR) n = 6	18.50 (15.50–21.75)	17.00 (6.50–18.75)	5.00 (−9.75 to 2.50)	0.143		
NSE +, median (IQR) n = 14	18.5 (15.75–23.25)	14.5 (7.75–18.0)	6.00 (−11.00 to 0.25)	0.013∗	0.856	0.913
NSE -, median (IQR) n = 7	20.00 (11.00–23.00)	12.00 (5.00–18.00)	6.00 (−14.00 to −1.00)	0.075		
**Peripheral blood eosinophilic (%)**						
Overall, median (IQR) n = 21	7.90 (4.30–10.70)	6.00 (3.55–7.70)	−1.70 (−4.00 to 0.55)	0.013∗	–	–
Male, median (IQR) n = 14	7.95 (3.30–11.18)	5.10 (3.58–6.78)	−1.60 (−4.25 to 0.48)	0.030∗	0.636	0.971
Female, median (IQR) n = 7	7.90 (5.00–10.70)	7.10 (3.30–8.50)	−2.40 (−4.30 to 2.10)	0.237		
sIgE +, median (IQR) n = 12	8.45 (4.48–10.68)	6.35 (3.15–9.18)	−2.35 (−4.15 to 0.23)	0.050	0.602	0.602
sIgE -, median (IQR) n = 9	5.80 (3.65–11.8)	6.00 (3.80–7.05)	−1.70 (−4.15 to 0.85)	0.139		
ECRSwNP, median (IQR) n = 16	9.20 (6.28–12.35)	6.70 (4.65–8.45)	−2.30 (−5.50 to −0.38)	0.011∗	<0.001∗∗∗	0.130
non-ECRSwNP, median (IQR) n = 5	3.00 (2.30–4.20)	3.50 (1.50–3.90)	0.40 (−1.90 to 0.85)	0.686		
NSE +, median (IQR) n = 14	8.45 (4.08–11.25)	6.00 (3.45–6.78)	−1.60 (−6.28 to 0.03)	0.011∗	0.535	0.322
NSE -, median (IQR) n = 7	5.00 (4.30–10.70)	7.10 (3.60–8.50)	−2.20 (−3.50 to 2.10)	0.398		
**Peripheral blood eosinophilic (count/** × **10**^**9**^**)**						
Overall, median (IQR) n = 21	0.50 (0.24–0.84)	0.46 (0.24–0.60)	−0.90 (−0.36 to 0.05)	0.019∗	–	–
Male, median (IQR) n = 14	0.51 (0.23–0.98)	0.34 (0.23–0.61)	−0.09 (−0.27 to 0.05)	0.035∗	0.856	NS
Female, median (IQR) n = 7	0.50 (0.24–0.84)	0.47 (0.28–0.60)	−0.14 (−0.48 to 0.10)	0.176		
sIgE +, median (IQR) n = 12	0.60 (0.24–0.83)	0.40 (0.23–0.62)	−0.09 (−0.41 to −0.02)	0.037∗	0.917	0.862
sIgE -, median (IQR) n = 9	0.36 (0.26–1.14)	0.46 (0.28–0.57)	−0.13 (−0.52 to 0.08)	0.139		
ECRSwNP, median (IQR) n = 16	0.70 (0.37–1.17)	0.48 (0.33–0.62)	−0.14 (−0.48 to −0.02)	0.011∗	<0.001∗∗∗	0.130
non-ECRSwNP, median (IQR) n = 5	0.21 (0.14–0.24)	0.23 (0.08–0.29)	0.04 (−0.09 to 0.08)	0.786		
NSE +, median (IQR) n = 14	0.66 (0.26–0.98)	0.41 (0.25–0.61)	−0.09 (−0.49 to 0.00)	0.017∗	0.488	0.400
NSE -, median (IQR) n = 7	0.35 (0.24–0.84)	0.46 (0.22–0.60)	−0.13 (−0.24 to 0.10)	0.310		
**NSE**						
Overall, n (Level 0/1/2/3/4) n = 22	7/4/9/2/0	16/6/0/0/0	7/9/5/1/0	<0.001∗∗∗	–	–
Male, n (Level 0/1/2/3/4) n = 14	2/3/9/0/0	9/5/0/0/0	2/8/4/0/0	0.001∗∗	0.407	0.447
Female, n (Level 0/1/2/3/4) n = 8	5/1/0/2/0	7/1/0/0/0	5/1/1/1/0	0.109		
sIgE +, n (Level 0/1/2/3/4) n = 12	3/3/4/2/0	8/4/0/0/0	3/6/2/1/0	0.006∗∗	0.539	0.722
sIgE -, n (Level 0/1/2/3/4) n = 10	4/1/5/0/0	8/2/0/0/0	4/3/3/0/0	0.024∗		
ECRSwNP, n (Level 0/1/2/3/4) n = 16	5/1/8/2/0	11/5/0/0/0	5/5/5/1/0	0.003∗∗	0.231	0.367
non-ECRSwNP, n (Level 0/1/2/3/4) n = 6	2/3/1/0/0	5/1/0/0/0	2/4/0/0/0	0.046∗		
NSE +, n (Level 0/1/2/3/4) n = 15	0/4/9/2/0	9/6/0/0/0	0/9/5/1/0	<0.001∗∗∗	<0.001∗∗∗	<0.001∗∗∗
NSE -, n (Level 0/1/2/3/4) n = 7	0/0/0/0/0	0/0/0/0/0	0/0/0/0/0	NS		

*P*_week 0–24_, the difference between week 0 and weeks 24. *P*_between groups baseline_, the difference between the baseline of the two groups. *P*_changes between 0–24_, the difference of the change in the two groups between week 0 and week 24. ACT, asthma control test; NPS, nasal polyp score; LMS, Lund-MacKay score; sIgE, specific IgE; ECRSwNP, eosinophilic chronic rhinosinusitis with nasal polyps; non-ECRSwNP, non-eosinophilic chronic rhinosinusitis with polyps; NSE, nasal smear eosinophilia; IQR, interquartile range; ∗*P* < 0.05; ∗∗*P* < 0.01; ∗∗∗*P* < 0.001; NS, not significant

### Objective clinical parameters outcomes

Twenty-one patients underwent sinus CT scan examinations at week 0 and week 24. The median change in LMS was 6.0 between the baseline and week 24 (*P* = 0.002) for all patients (Table 2). Further analysis revealed that the LMS significantly improved compared with baseline in the male subgroup (*P* = 0.016), the sIgE ±subgroups (*P* = 0.022 and *P* = 0.041, respectively), the ECRSwNP subgroup (*P* = 0.006) and the NSE + subgroup (*P* = 0.013); the female subgroup, non-ECRSwNP subgroup and the NSE-subgroup showed no reduction in the LMS (Table 2).

All patients achieved alleviation to varying degrees based on the nasal endoscopic examination. The LKS for nasal endoscopic examination showed significant improvement over several weeks [baseline-week 8 (*P* = 0.283), baseline-week 16 (*P* < 0.001), baseline-week 24 (*P* < 0.001)] (Table 3). The male subgroup (*P* < 0.001), the female subgroup (*P* < 0.001), the sIgE + subgroup (*P* < 0.001), the sIgE-subgroup (*P* < 0.001), the ECRSwNP subgroup (*P* < 0.001), the non-ECRSwNP subgroup (*P* = 0.010), the NSE + subgroup (*P* < 0.001) and the NSE-subgroup (*P* = 0.006) all showed a significant difference between baseline and week 24 (Table 3).

**Table 3:** Clinical characteristics of subjects at week 0, week 8, week 16 and week 24 of treatment.

	Week 0	Week 8	Week 16	Week 24	Change	*P*_*week*__0-8_	*P*_*week*__0-16_	*P*_*week*__0-24_	*P*_between groups baseline_	*P*_changes between 0-24_
**LKS**										
Overall, median (IQR) n = 22	12.00 (9.00–14.50)	10.00 (8.00–13.00)	8.50 (6.00–11.00)	7.00 (6.00–9.25)	−4.00 (−7.00 to −2.00)	0.283	<0.001∗∗∗	<0.001∗∗∗	–	–
Male, median (IQR) n = 14	13.50 (11.75–16.00)	10.00 (8.00–14.00)	10.00 (7.50–12.25)	7.50 (6.00–11.25)	−5.00 (−7.25 to −2.75)	0.404	0.006∗∗	<0.001∗∗∗	0.082	0.764
Female, median (IQR) n = 8	9.00 (8.00–13.75)	8.00 (7.25–12.25)	7.00 (3.75–9.50)	6.00 (3.75–7.50)	−4.00 (−7.00 to −2.25)	NS	0.030∗	<0.001∗∗∗		
sIgE +, median (IQR) n = 12	12.00 (9.00–15.50)	9.50 (8.00–13.75)	10.00 (6.25–11.75)	7.50 (6.00–10.50)	−4.00 (−7.00 to −3.00)	0.798	0.009∗∗	<0.001∗∗∗	0.974	NS
sIgE -, median (IQR) n = 10	12.00 (9.50–14.50)	10.00 (7.75–13.00)	8.00 (6.00–9.50)	6.50 (5.50–7.75)	−5.00 (−7.25 to −2.00)	NS	0.019∗	<0.001∗∗∗		
ECRSwNP, median (IQR) n = 16	12.00 (10.25–14.00)	10.00 (8.00–12.75)	8.50 (6.25–10.00)	7.00 (6.00–8.00)	−5.50 (−7.75 to −3.25)	0.522	0.001∗∗	<0.001∗∗∗	0.747	0.059
non-ECRSwNP, median (IQR) n = 6	11.50 (8.00–16.00)	10.50 (7.00–15.25)	10.00 (5.25–14.50)	8.50 (5.00–13.75)	−2.50 (−4.00 to −1.50)	NS	0.202	0.010∗		
NSE +, median (IQR) n = 15	12.00 (10.00–14.00)	10.00 (8.00–13.00)	8.00 (6.00–11.00)	7.00 (6.00–9.00)	−5.00 (−7.00 to −3.00)	0.286	0.001∗∗	<0.001∗∗∗	0.680	0.407
NSE -, median (IQR) n = 7	11.00 (9.00–16.00)	10.00 (8.00–13.00)	9.00 (6.00–11.00)	7.00 (6.00–10.00)	−3.00 (−7.00 to −2.00)	NS	0.137	0.006∗∗		

*P*_week 0-8_, the difference between week 0 and week 8. *P*_week 0-16_, the difference between week 0 and week 16. *P*_week 0-24_, the difference between week 0 and week 24. *P*_between groups baseline_, the difference between the baseline of the two groups. *P*_changes between 0-24,_ the difference of the change in the two groups between week 0 and week 24. LKS, Lund-Kennedy score; sIgE, specific IgE; ECRSwNP, eosinophilic chronic rhinosinusitis with nasal polyps; non-ECRSwNP, non-eosinophilic chronic rhinosinusitis with polyps; NSE, nasal smear eosinophilia; IQR, interquartile range; ∗*P* < 0.05; ∗∗*P* < 0.01; ∗∗∗*P* < 0.001; NS, not significant

All patients showed significant reduction in NPS between baseline and week 24 (*P* = 0.001). Furthermore, the male subgroup (*P* = 0.005), the female subgroup (*P* = 0.041), the sIgE + subgroup (*P* = 0.017), the sIgE-subgroup (*P* = 0.011), the ECRSwNP subgroup (*P* = 0.005), the non-ECRSwNP subgroup (*P* = 0.039), the NSE + subgroup (*P* = 0.005), and the NSE-subgroup (*P* = 0.038) all showed significant reduction (Table 2). Moreover, it could be noticed that the male subgroup, the ECRSwNP subgroup and the NSE + subgroup showing more observable improvement than that in the female subgroup, the sIgE-subgroup, the non-ECRSwNP subgroup, and the NSE-subgroup, respectively.

There was a decrease in the peripheral blood Eos% and peripheral blood Eos count (*P* = 0.013 and *P* = 0.019, respectively) between baseline and week 24 (Table 2). Interestingly, only the male subgroup, the sIgE + subgroup, the ECRSwNP subgroup and the NSE + subgroup showed significant decrease both in the peripheral blood Eos% and in the peripheral blood Eos count.

Regarding nasal smear eosinophilia, there was a prominent difference between week 0 and week 24 (*P* < 0.001). The sIgE±subgroup and the ECRSwNP/non-ECRSwNP subgroup showed improvement between the baseline and after the treatment of omalizumab. While the sIgE + subgroup (*P* = 0.006) and the ECRSwNP subgroup (*P* = 0.003) were more observable improvement than that in the sIgE-subgroup (*P* = 0.024) and the non-ECRSwNP subgroup (*P* = 0.046). Besides, the male subgroup (*P* = 0.001) showed the distinct amelioration, compared with the female subgroup (*P* = 0.109). We also found that NSE were less than level 1 in all patients after 24-week treatment of omalizumab (Table 2).

Twenty patients participated in PFTs at baseline and week 24. Although the ACT scores showed significant improvement in these patients, only the VC (actual/predicted) (*P* = 0.046), the PEF (actual/predicted) (*P* = 0.023), and the actual PEF (*P* = 0.032) showed significant amelioration between week 0 and week 24.

### ROC curve analysis

**Fig. 3:**
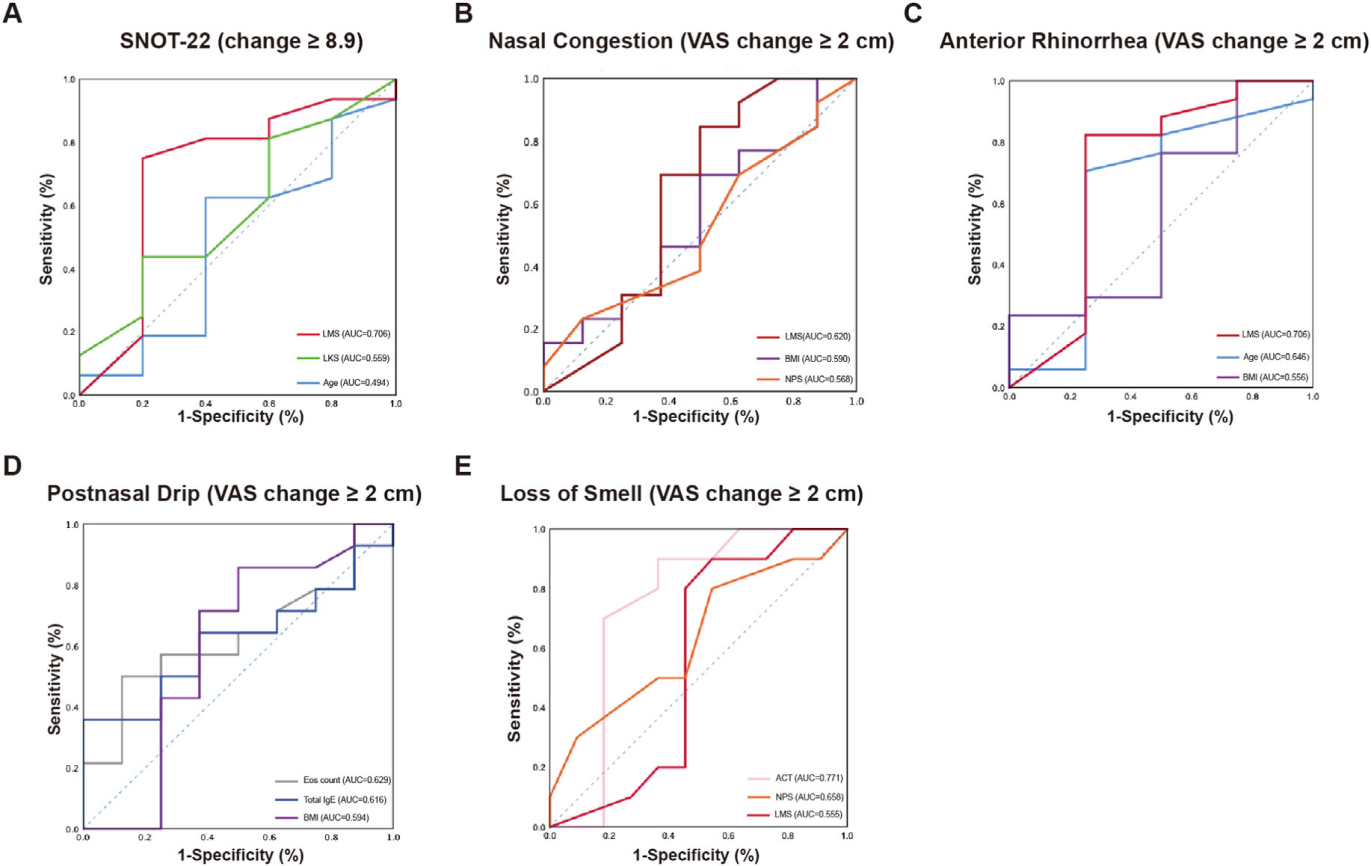
**Receiver operating characteristic (ROC) curve of clinical parameters to predict symptoms.** The peripheral blood eosinophilic (percent), peripheral blood eosinophilic **(**count/ × 10^9^) serum total IgE, ACT score, Age, BMI, LMS, LKS, and NPS as the indicator of the mean change in SNOT-22 scores (A), nasal congestion VAS reduction (B), anterior rhinorrhea VAS reduction (C), postnasal drip VAS reduction (D), and loss of smell VAS reduction (E) after treatment with omalizumab. We list the top 3 optimal indicators in each parameter and the area under the ROC curve (AUC) was shown at the right bottom.

ROC curves were used to analyze the predictive significance of the blood Eos (%), Eos (count), ACT scores, age, BMI, LMS, LKS and NPS for clinical symptom changes [SNOT-22 (Fig. 3A), nasal congestion VAS (Fig. 3B), anterior rhinorrhea VAS (Fig. 3C), postnasal drip VAS (Fig. 3D), and loss of smell VAS (Fig. 3E)] after treatment with omalizumab. An area of the predictor under the curve greater than 0.7 was acceptable for prediction. The details of acceptable predictors and the highest three Youden indexes are shown in Table 4. For the change in SNOT-22 (more than 8.9 points), the AUC of LMS was 0.706 (95% CI: 0.403–1.000), and the highest Youden index indicated that the optimal cutoff for LMS was 17.5 with a corresponding sensitivity and specificity of 75.0% and 80.0%, respectively. While the LMS cutoff value was 16.5 for predicting the reduction in anterior rhinorrhea VAS (more than 2 cm), with an AUC of 0.706 (95% CI: 0.338–1.000) (with the sensitivity: 82.4% and specificity: 75.0%). When the cutoff value of ACT was 18.5, the sensitivity and the specificity was 90.0% and 66.7% respectively, for the change of the loss of smell VAS (more than 2 cm), with an AUC of 0.771.

**Table 4:** The highest Youden indexes and the optimal cutoff value.

Predictor	AUC	95% CI	*P* value	Cutoff value	Sensitivity	Specificity	Youden Index
**SNOT-22 (change**≥**8.9)**							
LMS	0.706	0.403–1.000	0.173	***17.5***	75.0%	80.0%	0.550
**Anterior Rhinorrhea (VAS change**≥**2 cm)**							
LMS	0.706	0.338–1.000	0.210	***16.5***	82.4%	75.0%	0.574
**Loss of Smell (VAS change**≥**2 cm)**							
ACT	0.771	0.554–0.988	0.032	***18.5***	90.0%	66.7%	0.567

LMS, Lund-MacKay score; ACT, asthma control test; The bold and italic numbers indicate the best optimal cutoff value to predict. The *P* value indicates the difference between the ROC curve and the reference curve

## Discussion

**Fig. 4:**
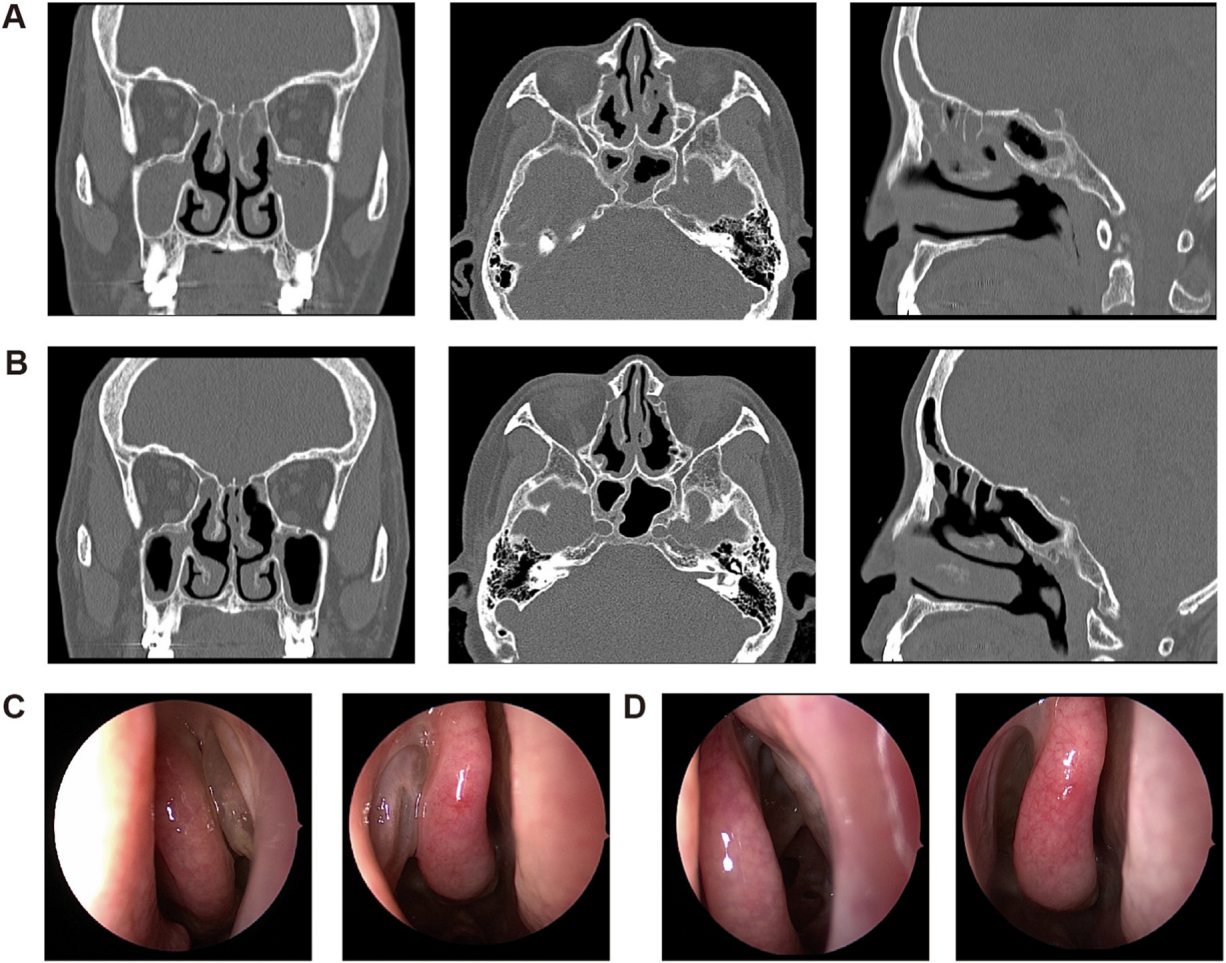
**Comparison of objective examination in one patient at week 0 and week 24.** A and C were the patients' CT scan and endoscopic pictures at baseline, and B and D were the patients' CT scan and endoscopic pictures after 24-week treatment with omalizumab.

In this research, only CRSwNP patients with simultaneous asthma could be recruited to comply with the registered indication of omalizumab specific for asthma by NMPA in China, which subsequently could be covered by social health insurance, considering its high cost. Meanwhile, asthma is also a very common comorbidity for CRSwNP patients, which easily leads to uncontrolled severe symptoms and frequent surgical failure and is prone to develop into refractory forms.^28^^,^^29^ Although EPOS 2020 recommended 1–2 short courses of systemic corticosteroids to alleviate uncontrolled CRSwNP, this could not become a routine medical choice due to its significant adverse effect and invalid heightening of QOL.^6^ Therefore, there is an urgent unmet demand to seek a novel treatment strategy for these recalcitrant CRSwNP patients to meet the clinical need for safety, efficacy, and long-term application. To our knowledge, we here conducted the first prospective study evaluating the therapeutic effect of omalizumab on difficult-to-treat CRSwNP patients with asthma in China and confirmed its beneficial outcome in ameliorating patients’ symptoms and QOL and improving CT and nasal endoscopy results (Fig. 4) and other clinical parameters by integrating subjective and objective evaluation methods.

The published data involving the effectiveness of omalizumab for the treatment of CRSwNP are insufficient and often contradictory. Both Gevaert and Tiotiu demonstrated a significant alleviation of nasal obstruction, rhinorrhea and loss of smell^17^^,^^30^ compared with another study showing no improvement in the SNOT-20 score or olfactory test results.^31^ Similarly, through endoscopic evaluations, Gevaert and Vennera separately found a significant decline in the total polyp score compared with placebo or baseline.^30^^,^^32^ In contrast, the other 2 studies reported an inapparent reduction in endoscopic polyp scores.^17^^,^^31^ Furthermore, by using simplified and qualitative methods, the latest study showed that omalizumab had a lower success rate of treatment in CRSwNP patients with asthma (50%) than mepolizumab had (78.9%).^26^ In this study, by adopting more complicated and comprehensive approaches, we noticed that the patients' VAS scores were significantly better, including nasal congestion, anterior rhinorrhea, postnasal drip, loss of smell, and the total SNOT-22 scores were evidently increased in all patients after 24 weeks of omalizumab therapy. Further objective evaluation also supported the positive efficacy of omalizumab. All patients’ median changes in LMS were 6 points, and both LKS and NPS showed significant improvement based on nasal endoscopic evaluation. Meanwhile, there was a significant decline in the peripheral blood Eos% and Eos count (from 7.9% to 6% and from 0.5 × 10^9^ to 0.46 × 10^9^, respectively) after 24-week therapy.

Although 86.36% of patients’ ACT scores exceeded 20 points, showing good asthma control levels, the results of only VC, PEF and actual PEF, but not FEV_1_, FVC and so on, achieved significant improvement from the pulmonary function data in this study, which also differed from previous studies. A single-center study showed that omalizumab significantly increased FEV_1_ (baseline: 1636 ± 628.4 mL) after 1 year (2000 ± 679.7 mL; *P* < 0.05) and 5 years (1929 ± 564.8 mL; *P* < 0.05) in 15 patients with severe allergic asthma.^33^ In another omalizumab study with a follow-up period of more than 27 months, both FEV_1_ and FVC were improved significantly in asthma patients with comorbid CRS and even more favorably than in asthma patients without CRS (*P* < 0.05).^28^ The inconsistency of the current results with the previous findings is probably attributable to the length of the treatment course. After all, this observational study on the efficacy of omalizumab lasted only 24 weeks. We have already found better subjective outcomes based on ACT scores and increased VC and PEF, reflecting beneficial changes in respiratory muscle strength. It is highly likely that more parameters of PFT, which are crucial and clinically significant, will be improved along with the prolongation of the treatment course.

Given the high cost of biological therapy and the fact that not all patients have good responses, the debate on its cost-effectiveness has been a focus in the long run. At present, there are still no clinical indicators of biomarkers that could effectively and accurately predict the efficacy of omalizumab. Therefore, it is particularly important to select the appropriate population for applying omalizumab to achieve personalized and accurate treatment. Encouragingly, some scholars have already made positive attempts to predict the response to anti-IgE strategies. The PROSPERO trial found that female asthma patients with a positive allergen-specific IgE result achieved more improvement 48 weeks after omalizumab initiation, according to one of three criteria: an annual exacerbation reduction ≥50%, improvement in ACT ≥20 points or increased FEV_1_ ≥ 120 mL. Moreover, patients with high blood Eos levels (≥300 cells/μL) were more likely to have improved ACT scores.^34^ Similarly, in severe allergic asthma patients after 48 weeks of omalizumab, the EXTRA study also demonstrated that the reductions in protocol-defined exacerbations were greater in the baseline level of the higher blood Eos subgroup (≥260 cells/μL), higher serum periostin subgroup (≥50 ng/mL) or higher fractional exhaled nitric oxide subgroup (≥19.5 ppb).^35^ In contrast, the STELLAIR study reported that the effectiveness of omalizumab was similar in high (≥300 cells/μL) and low (<300 cells/μL) blood Eos subgroups, which was assessed subjectively by five-point global evaluation of the treatment effectiveness scale, reduction of ≥40% in the annual exacerbation rate or a combination of both.^36^ However, the confounding factor, in which more than one-third of patients were still prescribed oral corticosteroids (average dose 20.4 mg/day), highly possibly contributed to the similarity of curative effects at both high and low Eos levels in the study. Compared with ample data involved in the prediction of the omalizumab response in asthma patients, predictive research specific for CRSwNP patients is rare and unsatisfactory. Recently, Meier has failed in identifying the predictive markers for successful therapy in CRSwNP patients treated with monoclonal antibody.^37^ In this study, we found that ECRwNP (blood Eos% > 4.27%) patients and male patients had better VAS (such as nasal congestion, loss of smell), SNOT-22 and ACT scores than non-ECRSwNP and female patients, respectively, when compared with baseline levels. Meanwhile, significant improvement in the LMS and NPS were observed among the ECRSwNP, male and NSE + subgroups after 24 weeks of omalizumab. Moreover, the remarkable decrease in both blood Eos% and Eos count were also found in the ECRSwNP, male, NSE+ and sIgE + subgroups. The multiple studies demonstrated asthma patients with high blood Eos levels could achieved significant improvement after omalizumab treatment.^34^^,^^35^ In terms of gender, female CRS patients might bear more severe disease burden. Lal pointed out that females suffered from worse QOL impairment on the basis of higher SNOT-22 scores than males did, although their CT scores were similar.^38^ Recently, one multicenter CRS study demonstrated female patients had worse postoperative QOL than male patients did, after experiencing standard clinical measures.^39^ By employing ROC analysis, we investigated the correlations between noninvasive parameters and some indicators for improvement acceptable to refractory CRSwNP patients suggested by EUFOREA and showed that a cutoff value of 17.5 and 16.5 in LMS had the moderate predictive value (AUC = 0.706) for the decline in the SNOT-22 score (more than 8.9 points) and reduction in anterior rhinorrhea VAS (more than 2 cm), respectively, which hinted the patients with more severe CT scores would probably achieve alleviation of subjective symptom and QOL after 24-week omalizumab treatment. In addition, a cutoff value of 18.5 in ACT could provide the moderate predictive value (AUC = 0.771) for the reduction of loss of smell VAS (more than 2 cm), which implied CRSwNP patients with mild asthma exacerbation were more likely to get better olfactory improvement after the application of omalizumab.

Although this prospective observational study avoided inherent selective biases originating from retrospective studies, there were also some limitations in the present study. First, the sample size was relatively small, which decreased the power of the research and limited the extent of the study achievement. Second, we failed to evaluate invasive biomarkers from nasal mucosa or polyps obtained by surgery or biopsy, considering that the endotype of disorders substantially affected the response to biological treatment.^28^ Third, we had to admit that our study is the lack of a placebo control arm, although it was the common shortcoming in some real-world studies. Subsequently, the results from this study need to be interpreted with caution.

## Conclusions

In this single-center study, we confirmed the beneficial effectiveness of omalizumab in Chinese patients with difficult-to-treat CRSwNP and asthma by employing a variety of minimally invasive or noninvasive evaluation methods, such as the VAS, SNOT-22, ACT, LMS, LKS, NPS, NSE, PFT, and routine blood tests. Moreover, better subjective and objective improvement could be found in ECRSwNP patients and in male patients than in other subgroup patients. Finally, multiple optimal cutoff values in the LMS and ACT had moderate predictive value for acceptable improvement after 24 weeks of omalizumab therapy. Future large-scale and multicenter studies combined with the detection of mucosal endotypes will potentially identify the etiology of discrepancies in outcomes and succeed in matching appropriate patients with omalizumab.

## Abbreviations

ACT, asthma control test; AUC, area under the receiver operating characteristic curve; BMI, body mass index; CRS, chronic rhinosinusitis; CRSsNP, chronic rhinosinusitis without nasal polyps; CRSwNP, chronic rhinosinusitis with nasal polyps; CT, computed tomography; ECRS, eosinophilic chronic rhinosinusitis; ECRSwNP, eosinophilic chronic rhinosinusitis with nasal polyps; EMA, European Medicines Agency; Eos%, eosinophilic percentage; EPOS, European Position Paper on Rhinosinusitis and Nasal Polyps; ESS, endoscopic sinus surgery; EUFOREA, European Forum for Research and Education in Allergy and Airway Diseases; FDA, Food and Drug Administration; FEV_1_, forced expiratory volume in 1 s; FVC, forced vital capacity; QOL, quality of life; HPF, high-power field; IgE, immunoglobulin E; LMS, Lund-MacKay score; LKS, Lund-Kennedy score; MCID, minimal clinically important difference; MEF_75_, maximum expiratory flow at 75% of vital capacity; MEF_50_, maximum expiratory flow at 50% of vital capacity; MEF_25_, maximum expiratory flow at 25% of vital capacity; NMPA, National Medical Products Administration; non-ECRS, non-eosinophilic chronic rhinosinusitis; non-ECRSwNP, non-eosinophilic chronic rhinosinusitis with nasal polyps; NPS, nasal polyp score; NSE, nasal smear examination; PEF, peak expiratory flow; PFT, pulmonary function test; ROC, receiver operating characteristic; sIgE, specific IgE; SNOT-22, 22-item sino-nasal outcome test; Th2, T helper 2; VAS, visual analogue scale; VC, vital capacity.

## Funding

The study was funded by the Changjiang Scholars and Innovative Research Team (IRT13082), the 10.13039/501100001809National Natural Science Foundation of China (81970852 and 82171110), the CAMS Innovation Fund for Medical Sciences (2019-I2M-5-022), and the Beijing Municipal Science and Technology Project (Z181100001618002), the Beijing Bai-Qian-Wan Talent Project (2019A32), the Public Welfare Development, Reform Pilot Project (2019–10), and the scientific research and cultivation fund of 10.13039/501100002799Capital Medical University (2020–1210020214).

## Availability of data and materials

The datasets supporting the conclusions of this study are available from the corresponding author on reasonable request.

## Authors’ contributions

MZ and YTSM conceptualized this study, analyzed, interpreted the data and draft the manuscript. LZ and XDW supervised and conceptualized the study and were involving in the important revision of the manuscript. XMW were responsible for the diagnosis of asthma. CYL, JMZ, SS and YW were involved in data collection. FFC and WX injected with omalizumab for patients. All authors read and approved the final version of the manuscript.

## Ethics approval and consent to participate

This study was approved by the Ethics Committee of Beijing TongRen Hospital, Capital Medical University (TRECKY2019-070) and Chinese Clinical Trial Registry (ChiCTR 1900026575). Informed consent was obtained from all participants.

## Authors’ consent for publication

All authors have read the final manuscript and consent for the submission and publication.

## Declaration of competing interest

All authors declare that they do not have any competing interests.

## Acknowledgements

Not applicable.
